# International comparison of physicians’ attitudes toward refusal of treatment by patients with anorexia nervosa: a case-based vignette study

**DOI:** 10.1186/s40337-022-00613-x

**Published:** 2022-06-23

**Authors:** Yoshiyuki Takimoto

**Affiliations:** 1 Department of Biomedical Ethics, Faculty of Medicine, The University of Tokyo, Tokyo, Japan; 2 Department of Psychosomatic Medicine and Stress Science, Faculty of Medicine, The University of Tokyo, Tokyo, Japan

**Keywords:** Anorexia nervosa, Treatment refusal, Compulsory treatment, Clinical ethics, Mental capacity, Decision-making

## Abstract

**Background:**

This study investigated the attitudes of physicians in Japan, the United Kingdom (UK), and the United States (US) toward refusal of treatment for anorexia nervosa.

**Methods:**

A questionnaire survey was administered to physicians treating patients with eating disorder (Japan, n = 55; UK, n = 84; US, n = 82) to evaluate their treatment strategies for fictitious cases of refusal of treatment for anorexia nervosa.

**Results:**

For acute patients, 53 (96.3%) physicians in Japan, 65 (77.4%) in the UK, and 54 (65.9%) in the US chose compulsory treatment if the patient’s family requested treatment, while 46 (83.6%) physicians in Japan, 53 (63.1%) in the UK, and 47 (57.3%) in the US chose compulsory treatment if the family left the decision to the patient. For severe and enduring anorexia nervosa, 53 (96.3%) physicians in Japan, 62 (73.8%) in the UK, and 57 (69.5%) in the US chose compulsory treatment if the patient’s family requested treatment, while 38 (69.1%) physicians in Japan, 56 (66.7%) in the UK, and 55 (67.1%) in the US chose compulsory treatment if the family left the decision to the patient.

**Conclusions:**

Physicians in all three countries tended to choose compulsory treatment irrespective of disease duration or whether the patient’s family requested treatment or not. This may indicate that medical practitioners value the ethical obligation of beneficence, giving priority to the protection of life. Attitudes toward refusal of treatment during a life crisis tend to vary among medical professionals, particularly if the patient’s family does not request treatment.

**Supplementary Information:**

The online version contains supplementary material available at 10.1186/s40337-022-00613-x.

## Plain English summary

This study aimed to investigate the attitudes of physicians in Japan, the UK, and the US toward refusal of treatment for anorexia nervosa. Physicians in all three countries received an anonymous questionnaire comprising four fictitious cases for which they had to respond whether they would choose compulsory inpatient treatment or not. The study revealed that in all three countries, compulsory treatment tended to be the prevalent choice in cases of life-threatening malnutrition, regardless of the patient's age or duration of illness. Moreover, in all the three countries, treatment tended to be forced if a family member requested treatment, and this trend was particularly strong in Japan. The influence of family request was stronger for acute AN in the U.K. and the U.S., and for SE-AN in Japan.

**Supplementary Information:**

The online version contains supplementary material available at 10.1186/s40337-022-00613-x.

## Background

Refusal of treatment can be a challenge for the treatment of eating disorders [1]. Obtaining informed consent from the patient is a prerequisite for performing certain therapeutic actions, such as hospitalization or nasogastric tube feeding, and the refusal of treatment by patients with eating disorders hampers treatment initiation. Particularly, refusal of treatment by low-weight patients with anorexia nervosa (AN) who need urgent medical treatment makes the management of such cases challenging. Furthermore, compulsory treatment needed to save a patient's life regardless of the patient’s wishes entails ethical concerns. For example, if a patient refuses treatment even when it is necessary, prioritizing the protection of life would infringe the patient's self-determination. Legal disputes and ethical debates have been raised on whether coercive treatment should be administered to patients with AN who refuse treatment. [2]. However, there are currently no guidelines or definitive opinions on the subject.

In Japan, the United Kingdom (UK), and the United States (US), patients with mental disorders who are at risk of self-injury or other harm can be legally and forcibly hospitalized, even if they do not consent to inpatient treatment [3]. In Japan, a system of hospitalization for medical protection allows treatment to be administered to a patient who is not in a condition to consent to treatment, even if there is no fear of self-injury or other harm; however, this is only done with the consent of the patient’s guardian [4]. This system of hospitalization for medical protection is different from those in Western countries, which emphasize the patient's right to self-determination, as underscored by the requirement for informed consent.

The attitudes of medical professionals toward refusal of treatment by patients with AN may possibly differ between Japan, the US, and UK due to differences in cultural [5] and legal [6] backgrounds. Various opinions have been expressed on the compulsory treatment of patients with AN who refuse treatment from the perspective of law and medical ethics [7]. However, the priorities for physicians and the attitudes they adopt when managing refusal of care by patients with life-threatening AN are unclear. Thus, this study aimed to evaluate the attitudes of expert physicians in Japan, the UK, and the US on refusal of treatment by patients with AN.

## Methods

An anonymous self-administered questionnaire survey was delivered by mail to 212 members of the Japanese Society for Eating Disorders, while an anonymous web-based questionnaire with similar questions created by double translation was administered to eating disorder specialists in the US and the UK. In the US, the web-based survey was conducted among physicians registered with MD.Linx (more than 415,000 doctors in total) who are members of eating disorder-related societies, such as the Academy of Eating Disorder and who practice eating disorder treatment. In the UK, the web-based survey was conducted among doctors registered with Doctors.net.uk (over 200,000 doctors in total) who are members of eating disorder-related societies, such as the British Eating Disorder Academy, and who practice eating disorder treatment. The web survey was conducted through a survey company that solicited responses until more than 80 responses were collected, assuming the maximum response rate in Japan was 40%. In both the US and the UK, three announcements encouraging cooperation in the survey were made over a six-week period.

Four fictitious vignette cases were used in the study, each comprising a combination of two different patient conditions (Case A and B) and two different reactions of the patients' families. The respondents were asked whether they would choose compulsory inpatient treatment or not (see Additional file 1). Case A is acute anorexia nervosa and Case B is severe and enduring anorexia nervosa defined by clinical severity, treatment failure or resistance, and chronicity [8]

### Statistical analysis

χ^2^ test was used to examine the differences in the physicians’ responses in the three countries. If the χ^2^ test result was significant, χ^2^ test or Fisher's direct method was used to analyze the differences in the responses between two countries, and Bonferroni’s correction was applied. *p* < 0.01 was considered statistically significant.

McNemar’s test was used to test the tendency of individual responses of physicians from each country to change between Case A and Case B and to test whether the individual responses changed depending on whether the patient’s family requested treatment or not.

All analyses were two-tailed and *p* value < 0.05 was considered statistically significant.

### Ethical considerations

This study was approved by the Ethics Committee of the Faculty of Medicine, The University of Tokyo (No. 3938-1).

## Results

### General characteristics of the respondents

Fifty-five valid responses were obtained from physicians in Japan who specialize in treating eating disorders (25.9% response rate). The physicians included 21 psychosomatic physicians, 24 psychiatrists, and 10 adolescent medicine physicians. Psychosomatic physicians are trained in internal medicine with additional psychiatric-psychosomatic trainings and both psychosomatic physicians and psychiatrists mainly treat eating disorders in Japan. Most physicians had 10 to 19 years of experience, while some had more than 30 years of experience. Most physicians treated 50 to 99 patients in a year, while some treated 150 to 199 patients in a year (Table 1).

**Table 1:** Characteristics of the respondents

	Years of experience as a clinician						
	< 5 years	5–9 years	10–19 years	20–29 years	> 30 years		
Japan (n = 55)	0	8	19	12	16		
UK (n = 84)	2	8	50	18	6		
US (n = 82)	4	17	36	20	5		

	Number of AN patients examined in a year						
	< 20 patients	20–49 patients	50–99 patient	100–149 patients	150–199 patients	200–299 patients	> 300 patients
Japan (n = 55)	0	8	19	12	16	0	0
UK (n = 84)	0	46	21	8	2	3	4
US (n = 82)	0	0	41	22	4	8	7

*UK* United Kingdom, *US* United States of America, *AN* anorexia nervosa

Eighty-four valid responses were obtained from physicians in the UK. All respondents were psychiatrists. Among the physicians who responded, 28.2% worked in clinics that specialized in treating eating disorders, 24.7% worked in hospitals that specialized in treating eating disorders, and 57.0% worked in other medical facilities. Most physicians had 10 to 19 years of experience, while some had 20 to 29 years of experience. Most physicians treated 20 to 49 patients for eating disorders per year, while some treated 50 to 99 per year.

Eighty-one valid responses were obtained from physicians in the US. All respondents were psychiatrists. Among the physicians who responded, 44.7% worked in clinics that specialized in treating eating disorders, 16.5% worked in hospitals that specialized in treating eating disorders, and 38.8% worked in other medical facilities. Most physicians had 10 to 19 years of experience, while some had 20 to 29 years of experience. Most physicians treated 50 to 99 patients per year, while some treated 100 to 149 patients per year.

The total number of samples from the three countries required for statistical analysis was 90, and this value was calculated by setting the difference at 40 points in accordance with previous studies [9], with α = 0.05 and β = 0.1 using POWER PROCEDURE of SAS.

### Comparison of responses from Japan, the UK, and the US

For young patients with acute AN, 53 (96%) physicians in Japan, 65 (77%) in the UK, and 54 (66%) in the US indicated that they would choose compulsory inpatient treatment if the patient’s family requested treatment. A significant bias was present in the response rates in the three countries. Bilateral comparison showed significant differences between the responses from Japan and those from the UK (*p* = 0.003) and between those from Japan and those from the US (*p* = 1.3 × 10^–4^) (Table 2).

**Table 2:** The proportion of physicians in Japan, the UK, and the US who chose involuntary inpatient treatment in four situations

	Acute patients with AN			Severe and enduring patients with AN		
	Patient’s family requested treatment	Patient’s family did not request treatment	McNemar’s test *P*-value	Patient’s family requested treatment	Patient’s family did not request treatment	McNemar’s test *P*-value
Japan (n = 55)						
CT	53 (96%)^ab^	46 (84%)^cd^	*p* = .125	53 (96%)^ef^	38 (69%)	*p* = 1.2 × 10^−4^
RW	2 (4%)	9 (16%)		2 (4%)	17 (31%)	
UK (n = 84)						
CT	65 (77%)^a^	53 (63%)^c^	*p* = .001	62 (74%)^e^	56 (67%)	*p* = .063
RW	19 (23%)	31 (37%)		22 (26%)	28 (33%)	
US (n = 82)						
CT	54 (66%)^b^	47 (57%)^d^	*p* = .022	57 (69%)^f^	55 (67%)	*p* = .022
RW	28 (34%)	35 (43%)		25 (31%)	27 (33%)	
χ^2^ test *P* value	χ^2^ = 16.987; df = 2; *p* = 2.1 × 10^−4^	χ^2^ = 14.656; df = 2; *p* = .005		χ^2^ = 16.556; df = 2; *p* = .002	χ^2^(2) = 0.565; df = 2; *p* = .754	

*CT* choice of compulsory inpatient treatment, *RW* respect for patient’s wishes, *UK* United Kingdom, *US* United States of America

^a^*p* = .003 by Fisher’s exact test

^b^*p* = 1.3 × 10^−4^ by Fisher’s exact test

^c^*p* = .008 by χ^2^ = 16.987; df = 1

^d^*p* = 4.9 × 10^−4^ by Fisher’s exact test

^e^*p* = .001, Fisher’s exact test

^f^*p* = 4.9 × 10^−4^ by χ^2^ = 15.703; df = 1

Forty-six (84%) physicians in Japan, 53 (63%) in the UK, and 47 (57%) in the US responded that they would choose compulsory inpatient treatment if the patient’s family left the decision to the patient. Additionally, a significant bias was detected in the response rates in the three countries. Bilateral comparison showed that there was a significant difference between the responses from Japan and those from the UK and between the responses from Japan and those from the US (Table 2).

For older patients with severe and enduring AN, 53 (96%) physicians in Japan, 62 (74%) in the UK, and 57 (70%) in the US indicated that they would choose compulsory inpatient treatment if the patient’s family wanted to initiate treatment. A significant bias was found in the response rates in the three countries. Bilateral comparison showed that there was a significant difference between the responses from Japan and those from the UK and between those from Japan and those from the US.

Thirty-eight (69%) physicians in Japan, 56 (66%) in the UK, and 55 (67%) in the US responded that they would choose compulsory inpatient treatment if the patient’s family left the decision to receive treatment to the patient. No significant bias was present in the response rates in the three countries.

### Comparison of response trends in each country

For young patients with acute AN, no difference was found between the tendency for individual physicians in Japan to choose compulsory treatment if the patient’s family wanted to initiate treatment and the tendency for them to choose compulsory treatment if the family left the decision to the patient. However, a significant difference was detected in the propensity of physicians in the UK and in the US to choose compulsory treatment (Table 2).

For older patients with severe and enduring AN, a significant difference was revealed between the tendency for individual physicians in Japan to choose compulsory treatment if the patient’s family wanted to initiate treatment and the tendency for them to choose compulsory treatment if the family left the decision to the patient. However, no significant difference was present in the tendency for physicians in the UK and the US to choose compulsory treatment.

No significant differences were found between the choices of physicians for young and older patients if family members requested treatment or if the patient’s family members left the decision to receive treatment to the patient.

### Comparison based on years of physician experience and number of case experiences

The differences in attitudes by years of experience and number of cases treated were examined among physicians in the UK and the US, where the trends were similar. No significant difference in attitude was found between physicians with more than 20 years of experience and those with less than 10 years of experience. Additionally, no significant difference in attitude was demonstrated between physicians who saw more than 100 cases per year and those who saw less than 30 cases per year (Table 3).

**Table 3:** Differences in attitudes based on years of physician experience and number of case experiences

	Patients with acute AN		Patients with severe and enduring AN	
	Patient’s family requested treatment	Patient’s family did not request treatment	Patient’s family requested treatment	Patient’s family did not request treatment
Physicians with less than 10 years of experience (n = 30)				
CT	21 (70%)	21 (70%)	17 (57%)	17 (57%)
RW	9 (30%)	9 (30%)	13 (43%)	13 (43%)
Physicians with at least 20 years of experience (n = 49)				
CT	34 (69%)	30 (61%)	36 (73%)	37 (76%)
RW	15 (31%)	19 (39%)	13 (27%)	12 (24%)
Fisher’s exact test *P* value	*p* = 1.00	*p* = .476	*p* = .144	*p* = .089
Physicians treating less than 30 cases of patients with AN per year (n = 37)				
CT	29 (78%)	22 (59%)	28 (76%)^f^	24 (65%)
RW	8 (22%)	15 (41%)	9 (24%)	13 (35%)
Physicians treating more than 100 cases of patients with AN per year (n = 57)				
CT	38 (67%)	36 (63%)	40 (70%)	40 (70%)
RW	19 (33%)	21 (37%)	17 (30%)	17 (30%)
Fisher’s exact test *P* value	*p* = .251	*p* = .829	*p* = .641	*p* = .653

*CT* choice of compulsory inpatient treatment, *RW* respect for patient’s wishes

## Discussion

This study was conducted to investigate the attitudes of physicians in Japan, the UK, and the US toward refusal of treatment for AN. To our knowledge, this is the first report of an international investigation on the propensity of physicians to choose compulsory treatment in cases of refusal of treatment for eating disorders. This study revealed that in Japan, the UK, and the US, compulsory treatment tends to be the prevalent choice in cases of life-threatening malnutrition, regardless of the patient's age or duration of illness. Compulsory treatment was chosen more often when family members requested treatment than when family members left the decision to the patient. The results also indicated that the tendency to choose compulsory treatment differed significantly among physicians in all the three countries.

Refusal of treatment in life-threatening cases of malnutrition poses the ethical dilemma of whether the physician should prioritize the protection of the patient's life or the patient's right to self-determination [10]. From the perspective of the four principles of medical ethics [11], it can be analyzed as an ethical issue of comparative consideration between the principle of good conduct and the principle of respect for autonomy. In the present study, physicians from Japan, the UK, and the US often chose the policy of compulsory treatment in life-threatening cases of poor nutrition, regardless of the patient's intention. The background of this attitude might be the idea of prioritizing the protection of life as the medical interest of the patient, which involves prioritizing the ethical obligation of beneficence over the ethical obligation of respect for autonomy [12–14].

There is no consensus on whether patients with AN are capable of making decisions regarding treatment [12, 15]. However, one reason that the duty of beneficence may take precedence over the patient's self-determination is the presumption that patients with AN who are undernourished are not in a condition to make sound decisions [16, 17]. In a situation where the patient cannot make appropriate decisions, it is common for family members to speculate on the patient's wishes on behalf of the patient. In fact, the responses in the present study indicated a greater tendency for the selection of compulsory treatment when the patient’s family wanted treatment than when they did not. This may be because the medical practitioner believes the patient is not competent enough to make sound decisions, and therefore, the physician follows the opinion of a family member who is the surrogate decision-maker. Conversely, over half of the physicians in Japan, the UK, and the US responded that they would choose compulsory treatment even if the patient’s family did not wish to initiate it. In such situations, it is probable that the medical care provider prioritized the patient’s best interest, which is protection of life, but the patient’s family did not consider the patient's best interest to be a priority [18]. In case of the refusal of treatment by patients who are experiencing life crises, Giordano [19] advocated for “legitimate use of prudence, recognition of the value of life, and common sense”, p 147 and stated that “if there is a fairly good chance that the patient will thank you for rescuing her, then you should rescue her.”, p 147. It has been reported that although patients with AN may refuse treatment, they are glad to have received coercive treatment after recovery [20]. However, this may be a strong form of paternalism [21].

The possibility of cognitive and affective biases in clinicians’ decision-making for patients with AN who refuse treatment cannot be ignored [22]. For example, self-serving concerns about the criticism by their colleagues and being sued for negligence may also be a factor in their conservative decisions (especially in the US) [23].

In the present study, the proportion of physicians who preferred involuntary treatment was significantly higher in Japan than in the US and the UK. This may be due in part to the fact that awareness of patient self-determination occurred late in Japan [24] and attitudes are more paternalistic, emphasizing the ethical obligation of beneficence [25]. The limited awareness of self-determination in Japan may be closely linked to Japanese culture, which is more family-centered than the more individualistic British and American cultures [26], and this may be another reason why the choice of compulsory treatment is prevalent in Japan, particularly if the patient’s family members request treatment. Moreover, Japanese law makes it easier to provide inpatient treatment when a patient refuses treatment despite having life-threatening malnutrition [4]. In Japan, a person who is not in a condition to be hospitalized voluntarily and is not at risk of self-injury or other harm can still be hospitalized after examination by a doctor and with family consent. The tendency for a patient’s family to influence the treatment plan in Japanese medicine has been reported previously [9].

In the present study, the number of physicians in the UK and the US who chose compulsory treatment for young patients was significantly lower when the decision was left to the patient than when the patient’s family wanted to initiate treatment. The reason for this may be that when the decision to initiate treatment is left to the patient, compulsory treatment may deteriorate the therapeutic relationship and make it difficult to continue treatment in the future. Although young patients with acute AN are less likely to be in mortal danger than are older patients with chronic AN [27], ensuring that treatment can be continued may be in their best interest. Furthermore, compulsory hospitalization may only have a limited therapeutic effect [28].

The differences between Cases A and B were in the patient’s age and disease duration. Case B was more severe because of the longer disease duration. Although some patients appreciate having received coercive treatment after recovery [20], some severely ill patients have been reported to oppose coercive treatment even after they have recovered [29]. For patients with severe AN with a long disease duration, the long-lasting fear of obesity may have been pathologically internalized as an identity; thus, refusing treatment may have reached the point of being a belief for the patients. In the present study, the proportion of physicians in all three countries who did not choose compulsory treatment for older patients with long disease duration was higher than that of physicians who did not choose compulsory treatment for younger patients. This trend of physicians not choosing compulsory treatment was also noted when family members left the decision to receive treatment to the patient; however, the result was not statistically significant. This trend may indicate that in the case of patients having severe and enduring AN with long disease duration, their morbid attitudes may have been interpreted as beliefs related to their identities. Thus, the physicians may have respected the patient's self-determination. Furthermore, even if a life-threatening AN crisis is averted for patients with severe and enduring disease, the crisis is likely to recur [30]. This may be a reason why some respondents in the present study considered involuntary treatment futile from a long-term perspective.

In the UK and the US, where the trends were relatively similar, attitudes toward refusal of treatment among physicians with many or few years of experience as clinicians and among physicians who saw many or few patients was examined, and no significant differences were found. Attitudes toward refusal of treatment reflect ethical values, and these results suggest that these values may be intrinsically present in each physician, rather than being developed by their experience as physicians.

### Strengths and limitations

Ethical issues in clinical practice are difficult to resolve with normative theory alone. When responding normatively to ethical issues in clinical practice, it is important to consider what should be done after understanding the current situation through empirical data. This is the first published international survey of physicians' attitudes toward refusal of treatment by patients with AN, and the data presented here could be used as reference when considering treatment refusal in patients with AN from the perspective of empirical bioethics [31]. However, this study has some limitations. First, the attitude toward a fictitious vignette case may differ from the attitude when confronted with an actual case of refusal of treatment in the course of clinical practice due to mental conflict. Second, the questionnaire was based on simulated cases; thus, the effect of the inpatient facilities and ethics policies of the respondents’ institutions on individual responses during actual cases was not reflected in this study. Third, owing to the small size of the study sample, the generalizability of the survey is difficult to estimate. Fourth, the response rate (40%) in Japan was lower than expected, possibly because a reminder was not sent to the participants. Fifth, since the survey was conducted using questionnaires delivered by mail in Japan, whereas web-based questionnaires were used instead in the UK and in the US, it is possible that differences in the survey method might have affected the results. Sixth, although a dual translation was used, the translation of the vignette cases may have altered some nuances, biasing the responses. Despite these limitations, this study is significant as it is the first published international survey conducted to compare the attitudes of medical practitioners toward refusal of treatment by patients with eating disorders.

In the future, it will be necessary to investigate whether physicians in practice recognize the mental capacity of patients with severe AN because mental capacity is directly related to the degree of demand to accept the patient's refusal of treatment. Additionally, an examination of how the mental capacity of patients with AN who refuse treatment is assessed and what type of treatment is provided when compulsory treatment is not chosen would also be helpful in the development of practical guidelines. Moreover, the degree of invasive treatment following compulsory hospitalization is a complex issue involving the principles of respect for autonomy, the principle of no harm, the principle of beneficence, and the law [2]. Therefore, it would make sense to investigate and analyze what is actually done in practice to guide decisions in case of refusal of treatment.

## Conclusions

Ideally, the response to refusal of treatment by patients with AN should be to protect life while respecting the patient's autonomy from an ethical standpoint. However, the approach for striking this balance is unclear. The present study revealed that the attitudes and choices of physicians toward refusal of treatment for anorexia nervosa tend to vary, particularly if the patient’s family members left the decision to receive treatment to the patient. Since the patient's condition and surrounding circumstances differ from case to case, it is difficult to determine a uniform response in advance. Therefore, the development of guidelines for shareable broad ethical ideas and decision-making procedures may be useful. Keeping the decision-making process uniform and fair, through guidelines, would satisfy procedural justice [32]. However, as demonstrated by the differences in the attitudes of physicians in each country in the present study, it is important to consider the background of each country while ensuring that the important ethical arguments are included in the guidelines. This is because guidelines based just on ethical idealism will be confusing in actual clinical practice. The issue of treatment refusal by patients with AN involves not only a clinical perspective but also ethical and cultural aspects. The results of this study may assist in examining multiple aspects of treatment refusal by patients with AN.

## Supplementary Information

**Additional file 1**. Vignette cases of patients with severe AN.

## Data Availability

The data that supports the findings of this study are available from the corresponding author on reasonable request.

## Abbreviations

UK: United Kingdom
US: United States
AN: Anorexia nervosa
