# Supplementary material for: International comparison of physicians’ attitudes toward refusal of treatment by patients with anorexia nervosa: a case-based vignette study
Source: J Eat Disord. 2022 Jun 23;10:86. doi: 10.1186/s40337-022-00613-x (PMC9230079; doi:10.1186/s40337-022-00613-x)
Supplement: Supplementary file 1 — Additional file 1. Vignette cases of patients with severe AN. [file 40337_2022_613_MOESM1_ESM.docx]

**APPENDIX**

CASE A:

Due to their nutritional condition being life-threateningly bad **(high risk of death due to malnutrition)** a **20-year-old** patient with an onset of anorexia nervosa **(developed six months prior with no previous treatment history at a medical institution)** comes to your hospital with their family members. However, despite the doctor’s advice, they have continued to refuse both admission and outpatient treatment saying “I do not want to receive any nutritional treatment, I do not want to get fat and I do not care even if my refusal leads to my death”. It might be possible to force treatment on them because they are not physically able to resist it due to them being weakened to the point where they have to stay in bed and cannot get up by themselves. There is no evidence that they have lost competence to judge.

QUESTION 1 Which decision do you make if the patient's family members wish for them to receive treatment?

QUESTION 2 Which decision do you make if the patient's family members wish for them to receive treatment?

CASE B:

Due to their nutritional condition being life-threateningly bad **(high risk of death due to malnutrition)** a **40-year-old** AN patient is in need of in-hospital treatment **(developed 24 years prior; has been brought to the hospital with the same condition several times, but has refused admission each time)** was brought to your hospital by their family members. However, despite the doctor’s advice they keep saying ”I do not want to receive any nutritional treatment, I do not want to get fat and I do not care even if my refusal leads to my death". It might be possible to force treatment on them because they are not physically able to resist it due to them being weakened to the point where they have to stay in bed and cannot get up by themselves. There is no evidence that they have lost competence to judge.

QUESTION 3 Which decision do you make if the patient's family members wish for them to receive treatment?

QUESTION 4 Which decision do you make if the patient's family members wish for them to receive treatment?
